# Assessment of the Turkish Version of the MMSE, MoCA, and ACE-R for Screening Mild Vascular Cognitive Impairment

**DOI:** 10.3390/healthcare14193234

**Published:** 2026-10-01

**Authors:** Zeliha Yücel, Merve Akgül Günay

**Affiliations:** Department of Neurology, School of Medicine, Karamanoglu Mehmetbey University, 70100 Karaman, Turkey

**Keywords:** vascular cognitive impairment (VCI), Standardized Mini-Mental State Examination (MMSE), Addenbrooke’s Cognitive Examination Test (ACE-R), cognitive screening

## Abstract

**Aim**: Vascular cognitive impairment (VCI), which affects many people, is important to distinguish from other types of cognitive impairment because vascular risk factors can slow disease onset and progression. In our study, we aimed to determine the most appropriate cognitive screening tool for vascular cognitive impairment. **Methods**: This study included 110 patients with mild VCI and a control group of 90 volunteers aged over 55 and was conducted between July 2024 and September 2025. Mild VCI was diagnosed according to DSM-5 criteria. We administered the Montreal Cognitive Assessment (MoCA), the Standardized Mini-Mental State Examination (MMSE), and Addenbrooke’s Cognitive Examination Test (ACE-R) to all participants. **Results**: Our findings demonstrate that all three screening tools significantly differentiate patients with mild VCI from healthy controls. ACE-R had the highest area under the curve (AUC = 0.967) and showed 90% sensitivity and 100% specificity at a cut-off of 68.5. MoCA also showed relatively high sensitivity (86.4%) and specificity (91.1%), with high diagnostic accuracy (AUC = 0.946). In contrast, the MMSE showed a slightly lower diagnostic performance (AUC = 0.926) but still had high specificity (92.2%). **Conclusions**: Our study demonstrates that the ACE-R is the most accurate screening tool for detecting mild vascular cognitive impairment, with the MoCA trailing by a very small margin, while the MMSE may be less sensitive in the early stages. Given the administration time, the MoCA can be a quicker alternative to the ACE-R.

## 1. Introduction

Vascular cognitive impairment (VCI) refers to cognitive changes associated with cerebrovascular disease. VCI is a heterogeneous group of disorders spanning the clinical spectrum from vascular mild cognitive impairment to vascular dementia (VaD) [1]. Currently, VaD is the second most common type of dementia after Alzheimer’s disease, with an estimated prevalence of 324 per 10,000 people [2,3]. The prevalence of cerebrovascular disease is increasing, and VCI is expected to rise accordingly [4]. VCI is heterogeneous, and general-purpose cognitive screening tools were not designed for this condition [5]. Most widely used tools were developed and validated for Alzheimer’s-type diseases, which are typically characterized by memory deficits and cortical involvement. In contrast, VCI manifests primarily as executive dysfunction, slowed processing speed, and attention problems; a tool optimized to detect amnestic decline may result in VCI cases being overlooked [6,7]. It is important to distinguish VCI from other types of cognitive impairment because identification and treatment of vascular risk factors, particularly hypertension, have been shown to slow disease onset and progression [8]. Various studies have investigated the Montreal Cognitive Assessment (MoCA), Standardized Mini-Mental State Examination (MMSE), Short Memory and Executive Functions Test (BMET), Frontal Assessment Battery (FAB), Clock Drawing Test (CDT), Trail Making Test (TMT), and Addenbrooke’s Cognitive Examination Test (ACE- R) as screening tools for VCI. Still, no consensus has been found on the most appropriate cognitive screening tool [9]. Therefore, we thought a comparative study of VCI-specific cognitive screening tools would make a valuable contribution to the literature. Our study aimed to investigate the specificity and sensitivity of cognitive screening tools, including the Montreal Cognitive Assessment (MoCA), the Standardized Mini-Mental State Examination (MMSE), and Addenbrooke’s Cognitive Examination-R (ACE-R), and to determine the most appropriate screening tool.

## 2. Material and Methods

### 2.1. Study Design

This study was conducted at the Karaman Training and Research Hospital Neurology Clinic, and we enrolled participants between July 2024 and September 2025. The Karamanoglu University Ethics Committee approved the study protocol per the ethical standards outlined in the Declaration of Helsinki (IRB number 11095095-050.04-195373). All participants were informed of the possible risks and benefits, and we obtained written informed consent.

### 2.2. Participants

This study included patients diagnosed with vascular cognitive impairment at the Neurology Clinic of Karaman Training and Research Hospital. Patients over 55 years of age who presented to the Neurology Clinic with cognitive complaints and a history of cerebrovascular disease underwent neurological examination, cranial magnetic resonance imaging (MRI), and tests for B12, folate, iron deficiency, and thyroid function tests.

Patients are divided into two groups: mild and major vascular cognitive impairment (VCI). The diagnosis of mild and major VCI is determined according to DSM-V criteria. These criteria encompass mild and major vascular cognitive impairment. The criteria consist of the following:

1. Presence of clinical findings suggestive of a vascular etiology: the onset of cognitive deficit must be temporally linked to one or more cerebrovascular events or a significant reduction in frontal executive functions and complex attention, including processing speed, must be present.
2. Supported by history, physical examination, and/or imaging studies of a cerebrovascular disease thought to be responsible for the neurocognitive deficit.
3. Another brain disease or systemic disorder cannot better explain the symptoms. The distinction between mild and major VCI is determined by the presence or absence of impairment in activities of daily living. No impairment in activities of daily living is considered mild VCI, while impairment in activities of daily living is considered major VCI. We excluded patients with major VCI from the study. We included 110 patients with mild VCI.

The control group consisted of volunteers aged 55 years or older with no cognitive complaints and no vascular gliotic foci on MRI; we included 90 volunteers.

### 2.3. Neuropsychological Assessment

Cognitive functions were assessed in all participants using the Turkish ACE-R, MoCA, and MMSE. Overlapping items in different cognitive tests (e.g., clock drawing or specific questions related to orientation) were administered only once. To avoid bias, all cognitive screening assessments were administered by two neurology specialists who were blind to grouping. The evaluators administering the test were unaware of the patient’s scores on other tests.

The ACE-R consists of five domains, each reflecting a specific cognitive function and contributing equally to the total score. The total score is 100 points, distributed across the five cognitive domains as follows: 18 points for attention and orientation, 26 points for memory, 14 points for fluency, 26 points for language, and 16 points for visual–spatial.

The MoCA is a 30-point scale consisting of 7 cognitive subtests: 5 points for visual–executive, 3 points for naming, 6 points for attention, 3 points for language, 2 points for abstraction, 5 points for delayed recall, and 6 points for orientation.

The Standardized MMSE is a 30-point scale consisting of 5 cognitive subtests: 10 points for orientation, 6 points for memory, 5 points for attention, 8 points for language, and 1 point for visual–executive.

### 2.4. Statistical Analysis

The sample size was calculated based on a comparison of cognitive test scores between two independent groups. A medium effect size (Cohen’s d = 0.50) was assumed. A power analysis—conducted with a Type I error rate of α = 0.05 and a test power of 90%—determined that a minimum of 172 participants were required, with at least 86 participants in each group. This study included 200 participants: 110 patients and 90 controls. We assessed the normality of the continuous variables graphically and with the Shapiro–Wilk test. Descriptive statistics were presented as mean ± standard deviation and median (IQR; 25th–75th percentile). We created cross-tables to compare variables by Patient-Control group and reported the number (*n*), percentage (%), and chi-square test statistics. We used the Mann–Whitney U test to compare MMSE, MoCA total, and MoCA delayed recall score.

To account for potential confounding effects arising from age differences between groups and to evaluate sex as a factor, we performed a two-factor analysis of covariance (ANCOVA) for each cognitive score. The model included the group (patient and control) and sex (female and male) as fixed factors and age as a continuous covariate; the group-by-sex interaction was also tested. We reported adjusted differences and adjusted Cohen’s d values between patients and controls, along with 95% confidence intervals. For results showing a significant group-by-sex interaction, sex-specific adjusted comparisons were also presented.

ACE-R total score, ACE-R Attention, ACE-R Memory, ACE-R Verbal Fluency, ACE-R Language, and ACE-R Visual–Executive values are according to the Patient-Control group. Spearman’s nonparametric correlation coefficient was reported in the correlation analyses. ROC (Receiver Operating Characteristic) analysis was performed using the Patient-Control grouping reference for clinically predicted MMSE, MoCA total, MoCA delayed recall score, ACE-R total score, ACE-R Attention, ACE-R Memory, ACE-R Verbal Fluency, ACE-R Language, and ACE-R Visual–Executive values that may be effective in identifying the at-risk group, and the ROC graph was drawn; the area under the curve (AUC) and its 95% confidence intervals were determined, and sensitivity and specificity values were given. IBM SPSS Statistics 26.0 (IBM Corp., released 2019. IBM SPSS Statistics for Windows, Version 26.0. Armonk, NY, USA: IBM Corp.) and Microsoft Excel 2024 were used for statistical analyses and calculations.

## 3. Results

By Patient-Control grouping, age differed significantly (t = 2.868, *p* = 0.005). The average age of individuals in the patient group was higher than in the control group. Between the patient and control groups, no statistically significant differences were found in gender or education status (*p* > 0.05) (Table 1).

The DeLong test revealed a statistically significant difference in performance between MMSE and ACE-R total scores (*p* = 0.021). The ACE-R total score is better than the MMSE in distinguishing between patients and controls. Furthermore, the DeLong test showed no statistically significant performance difference between MMSE-MoCA Total and MoCA Total-ACE-R total scores (*p* > 0.05) (Table 2).

By Patient-Control group, we found statistically significant differences in MMSE, MoCA total, MoCA delayed recall score, ACE-R total score, ACE-R Attention, ACE-R Memory, ACE-R Verbal Fluency, ACE-R Language, and ACE-R Visual–Executive values (*p* < 0.001). The mean MMSE, MoCA total, MoCA delayed recall score, ACE-R total score, ACE-R Attention, ACE-R Memory, ACE-R Verbal Fluency, ACE-R Language, and ACE-R Visual–Executive values were lower in the patient group than in the control group (Table 3).

We found a very high, positive, statistically significant correlation between MMSE and MoCA Total, MoCA Delayed Recall score, and ACE-R total score (r = 0.858; *p* < 0.001, r = 0.843; *p* < 0.001, r = 0.841; *p* < 0.001). A high-level, positive, statistically significant correlation was found between MMSE and ACE-R Attention, ACE-R Memory, ACE-R Verbal Fluency, and ACE-R Language values (r = 0.695; *p* < 0.001, r = 0.774; *p* < 0.001, r = 0.613; *p* < 0.001, r = 0.620; *p* < 0.001). A moderate, positive, statistically significant correlation was found between MMSE and ACE-R Visual–Executive values (r = 0.591; *p* < 0.001).

A very high, positive, statistically significant correlation was found between MoCA Total and ACE-R total score and ACE-R Memory values (r = 0.871; *p* < 0.001, r = 0.826; *p* < 0.001). A high level of positive statistically significant correlation was found between MoCA Total and MoCA Delayed recall score, ACE-R Attention, and ACE-R Verbal Fluency, ACE-R Language, ACE-R Visual–Executive values (r = 0.768; *p* < 0.001, r = 0.696; *p* < 0.001, r = 0.674; *p* < 0.001, r = 0.613; *p* < 0.001, r = 0.657; *p* < 0.001). We found a statistically significant, positive correlation between MoCA Delayed Recall Score and ACE-R total score and ACE-R Memory values (r = 0.731; *p* < 0.001, r = 0.685; *p* < 0.001). A statistically significant, positive correlation was found between MoCA Delayed Recall Score and ACE-R Attention, ACE-R Verbal Fluency, ACE-R Language, and ACE-R Visual–Executive values (r = 0.580; *p* < 0.001, r = 0.522; *p* < 0.001, r = 0.502; *p* < 0.001, r = 0.509; *p* < 0.001) (Table 4).

As a result of the ROC analysis performed with the Patient-Control group as a reference, MMSE, MoCA total, MoCA delayed recall score, ACE-R total score, ACE-R Attention, ACE-R Memory, ACE-R Verbal Fluency, ACE-R Language, and ACE-R Visual–Executive curve areas were found to be statistically significant (*p* < 0.001). Using the Patient-Control group as the reference, the cut-off point for the MMSE variable was 25.5. For MMSE ≤ 25.5, sensitivity was 82.70%, and specificity was 92.20%. Using the Patient-Control group as the reference, the cut-off point for the MoCA Total variable was 22.5.

For MoCA Total ≤ 22.5, sensitivity was 86.40%, and specificity was 91.10%. The ACE-R total score, calculated using patient-control grouping as a reference, had a cut-off point of 68.5. For ACE-R total score ≤ 68.5, sensitivity was 90.00% and specificity was 100.00% (Table 5).

After adjusting for age and gender, the group main effect remained significant across all cognitive measures examined (*p* < 0.001). Adjusted mean differences for the patient group were −3.17 points for the MMSE, −5.86 points for the MoCA Total, and −20.63 points for the ACE-R Total Score; the absolute adjusted standardized differences for these three main tests were 2.11, 2.16, and 3.15, respectively. The age covariate was found to be significant for all outcomes (*p* < 0.05). While the main effect of gender was significant for all measures except ACE-R Memory, the group-by-gender interaction was significant for all measures except ACE-R Memory and ACE-R Verbal Fluency.

It was determined that 61.9% (*n* = 13) of those classified as Cranial MRI-Fazekas I had MMSE ≤ 25.5 and 38.1% (*n* = 8) had MMSE > 25.5; 85.0% (*n* = 34) of those classified as Cranial MRI-Fazekas II had MMSE ≤ 25.5 and 15.0% (*n* = 6) had MMSE > 25.5; and 89.8% (*n* = 44) of those classified as Cranial MRI-Fazekas III had MMSE ≤ 25.5 and 10.2% (*n* = 5) had MMSE > 25.5. Among individuals classified as Cranial MRI-Fazekas I, 90.5% (*n* = 19) had a MoCA Total score of 22.5 or lower, while 9.5% (*n* = 2) had a score higher than 22.5. For those classified as Cranial MRI-Fazekas II, 85.0% (*n* = 34) had a MoCA Total score of 22.5 or lower, and 15.0% (*n* = 6) scored above 25.5. Additionally, among individuals classified as Cranial MRI-Fazekas III, 85.7% (*n* = 42) had a MoCA Total score of 22.5 or lower, whereas 14.3% (*n* = 7) scored above 25.5. It was determined that 85.7% (*n* = 18) of those classified as Cranial MRI-Fazekas I had an ACE-R total score ≤ 68.5, and 14.3% (*n* = 3) had a score > 68.5; 82.5% (*n* = 33) of those classified as Cranial MRI-Fazekas II had an ACE-R total score ≤ 68.5, and 17.5% (*n* = 7) had a score > 68.5; and 98.0% (*n* = 48) of those classified as Cranial MRI-Fazekas III had an ACE-R total score ≤ 68.5, and 2.0% (*n* = 1) had a score > 68.5 (Table 6).

## 4. Discussion

In our study, although ACE-R stood out among the three screening tools (SS, MoCA, and ACE-R), we found a significant difference between MMSE and ACE-R (*p* = 0.021), while MoCA and ACE-R showed no statistically significant difference in total scores (*p* > 0.05). One of the most significant findings is that the ACE-R total score has the highest area under the curve (AUC = 0.967) and showed 90% sensitivity and 100% specificity at a cut-off of 68.5. MoCA also demonstrated high diagnostic accuracy (AUC = 0.946), with relatively high sensitivity (86.4%) and specificity (91.1%). In contrast, MMSE showed a slightly lower diagnostic performance (AUC = 0.926) but still had high specificity (92.2%). This suggests that the ACE-R may be the most precise screening instrument for identifying mild vascular cognitive impairment (VCI) in our cohort. This finding may stem from ACE-R’s ability to better capture the heterogeneous cognitive profile of vascular cognitive impairment, given its multi-domain structure (attention, memory, fluency, language, and visuospatial abilities). Furthermore, the ACE-R memory subtest incorporates the three-word registration/recall test found in the MMSE; in other words, it not only encompasses the MMSE but also extends its scope. Unlike the MMSE, which utilizes a simple six-point registration/recall format, the ACE-R includes semantic memory items as well as a multi-trial name-and-address learning, recall, and recognition paradigm. Although frontal lobe dysfunction is primarily expected in VCI patients, VCI is a heterogeneous condition encompassing multiple clinical syndromes [10] and arising from various pathological mechanisms [11]. This heterogeneity likely explains why an instrument such as MMSE, which allocates minimal weighting to visuospatial and executive function, is comparatively insensitive to the cognitive signature of VCI, whereas ACE-R’s balanced multi-domain structure is better positioned to capture impairment regardless of which cognitive domain predominates in a given patient.

The literature regarding screening accuracy for vascular cognitive impairment has primarily compared the MoCA and MMSE rather than the ACE-R [12,13,14,15]. In a study by Pendlebury et al. comparing these screening tools, the ACE-R and MoCA performed similarly, and neither tool showed clear superiority. However, our study found a statistically significant difference between the MMSE and the ACE-R (AUC = 0.967 vs. 0.946; *p* = 0.021). This discrepancy likely stems from differences in study populations: Pendlebury et al. examined patients with cerebrovascular disease at least one year after a stroke or transient ischemic attack (TIA), whereas our study used DSM-5 diagnostic criteria for vascular cognitive impairment (VCI) [11]. A 2018 systematic review and meta-analysis evaluating the MoCA for post-stroke cognitive impairment—covering 12 studies and over 2100 patients—reported that the MoCA demonstrated significantly higher sensitivity than the MMSE but performed similarly to the ACE-R [16]. Similarly, a systematic review evaluating the sensitivity and specificity of post-stroke cognitive screening tools found that the MoCA had a more favorable profile than the MMSE; however, including the ACE-R in only one reviewed study limited direct comparisons [17]. In contrast, our finding that the ACE-R performs better than the MoCA contrasts with the systematic review by Ghafar et al. (2019) [17], which evaluated eight cognitive screening tools and identified the MoCA—demonstrating good reliability and internal consistency—as the most accurate tool for detecting VCI. Several factors may explain this discrepancy. First, while the results of Ghafar et al. are based on a synthesis of numerous heterogeneous studies employing varying reference standards, VCI definitions, and populations, our findings derive from a single-center, direct comparison conducted within a single cohort using consistent DSM-5 diagnostic criteria; although this study design may better reveal the true comparative performance, it limits the generalizability of the results. Second, the populations included in the study by Ghafar et al. may have disproportionately represented individuals with more advanced stages of VCI or post-stroke VCI; in such clinical settings, the shorter MoCA test—which focuses on subcortical–executive functions—tends to excel. In contrast, our cohort study focused on mild VCI cases; at this stage, the ACE-R’s broader, multi-domain nature (including memory) may offer added value in discriminative ability [17]. The relative scarcity of ACE-R data in the post-stroke or VCI screening literature [12,13,14,17] highlights our study’s unique contribution: a direct comparison of all three tools in a cohort of individuals diagnosed with mild VCI according to DSM-5 criteria.

Our study also revealed a strong, positive correlation among MMSE, MoCA, and ACE-R scores. Although these screening tests differ in structure and sensitivity, their correlation indicates that they assess overlapping cognitive domains. Particularly strong correlations were observed between the MoCA and ACE-R in the domains of memory and executive function; this may explain their similar diagnostic utility. On the other hand, while the ACE-R demonstrated the highest diagnostic accuracy, it required the longest administration time (~15–20 min) compared to the MoCA (~10–15 min) and MMSE (~5–10 min)—a factor to consider when selecting a screening tool for time-constrained clinical settings [18]. These findings further suggest that the MoCA could serve as a quicker alternative to the ACE-R in busy clinical environments, whereas the ACE-R might be preferred when a more detailed cognitive profile is required.

Subgroup analysis showed that the ACE-R memory domain (AUC = 0.997) had the greatest discriminatory power. This is an interesting finding, as vascular cognitive impairment is traditionally associated with executive function impairment; however, this suggests that memory impairment may also play a significant role in mild VCI or may reflect mixed pathology in this patient population. In addition, MoCA showed high specificity (97.8%) for delayed recall, suggesting that it may be particularly useful for diagnosing cognitive impairment when delayed recall is abnormal [19]. Differences in subgroup scores and their distribution may also have affected statistical significance.

The Fazekas scale, proposed by Fazekas et al. in 1987 [20], measures the amount of white matter T2 hyperintense lesions, commonly attributed to chronic small vessel ischemia. The Fazekas grading system further supported the clinical significance of the screening tools evaluated in our study. As white matter lesion severity increased, the proportion of patients with SS, MoCA, and ACE-R scores below the cut-off values also increased. Previous studies in the literature also support our study [21,22,23]. A recent meta-analysis including 15 studies with a total of 4061 patients showed that the severity of vascular dementia increased as the WMH burden increased [24]. This suggests a link between structural brain changes and cognitive decline, consistent with the known pathophysiology of vascular cognitive impairment. Among the tools used, ACE-R demonstrated the most consistent performance with the Fazekas grading, further supporting its reliability.

Our study has several limitations. First, the age difference between the patient and control groups may have affected the results, since age is a known factor that influences cognitive performance. Second, this study was conducted at a single center with a relatively small sample size, which may limit its broader applicability. Third, we included only patients with mild vascular cognitive impairment; therefore, the findings may not generalize to vascular dementia. Furthermore, although the educational level did not differ statistically between groups, it may have influenced the test performance.

## 5. Conclusions

Our study shows that ACE-R is the most accurate screening tool for detecting mild vascular cognitive impairment, closely followed by MoCA, while MMSE, though useful, may be less sensitive in the early stages. Although ACE-R is the most accurate screening tool for detecting mild vascular cognitive impairment, MOCA offers a good balance of sensitivity, specificity, and practicality, making it a strong candidate for routine clinical screening. Further studies involving larger, multicenter cohorts and different subtypes of vascular cognitive impairment are needed to validate these findings further and establish standardized screening recommendations.

## Figures and Tables

**Table 1:** Demographic characteristics.

		Patients (*n* = 110)	Controls (*n* = 90)	Statistic	
				t; χ^2^	*p*
Age (year)	Mean ± SD Median (IQR)	71.85 ± 7.08	69.39 ± 5.04	t = 2.868	0.005
		72.0 (68–76)	69.5 (66–73)		
**Gender, *n* (%)**					
Female		58 (52.7)	45 (50.0)	$\chi^{2}$ = 0.147	0.701
Male		52 (47.3)	45 (50.0)		
**Educational Status**					
Primary school		78 (70.9)	63 (70.0)	$\chi^{2}$ = 0.020	0.888
High school or above		32 (29.1)	27 (30.0)		

t: Independent-samples *t*-test, $\chi^{2}$: Chi-square test.

**Table 2:** Comparison of MMSE, MoCA, and ACE-R in determining mild VCI.

	MMSE	MoCA Total	ACE-R Total Score
	*p*	*p*	*p*
**MMSE**		0.208	**0.021**
**MoCA Total**	0.208		0.232
**ACE-R total score**	**0.021**	0.232	

**Table 3:** Comparison of clinical values according to patient-control grouping.

	Patients (*n* = 110)		Controls (*n* = 90)		Statics	
	Mean ± SD	Median (IQR)	Mean ± SD	Median (IQR)	z	*p*
**MMSE**	23.77 ± 2.02	24.0 (22–25)	27.24 ± 1.09	27.0 (27–28)	z = 10.477	**<0.001**
**MoCA Total**	18.42 ± 3.65	18.0 (16–21)	24.67 ± 1.49	25.0 (24–26)	z = 10.911	**<0.001**
**MoCA Delayed Recall Score**	2.28 ± 0.96	2.0 (2–3)	3.82 ± 0.77	4.0 (3–4)	z = 9.673	**<0.001**
**ACE-R Total Score**	55.05 ± 9.49	55.0 (47–60)	77.27 ± 4.33	77.0 (74–79)	z = 11.359	**<0.001**
**ACE-R Attention**	12.72 ± 2.77	13.0 (11–14)	15.51 ± 1.28	16.0 (14–16)	z = 7.592	**<0.001**
**ACE-R Memory**	7.10 ± 3.16	7.0 (5–9)	19.36 ± 2.02	19.0 (18–20)	z = 12.155	**<0.001**
**ACE-R Verbal Fluency**	5.35 ± 3.17	6.0 (3–7)	9.52 ± 1.28	10.0 (8–10)	z = 9.171	**<0.001**
**ACE-R Language**	15.96 ± 4.79	16.0 (13–19)	19.19 ± 2.42	19.0 (18–20)	z = 5.615	**<0.001**
**ACE-R Visual–executive**	11.13 ± 3.02	11.0 (8–14)	13.93 ± 1.22	14.0 (13–14)	z = 6.854	**<0.001**

z: Mann–Whitney U Test.

**Table 4:** Relationship between MMSE, MoCA Total, MoCA Delayed Recall Score, and ACE-R Values.

	MMSE		MoCA Total		MoCA Delayed Recall Score	
	r	*p*	r	*p*	r	*p*
**MMSE**	-	-	0.858	**<0.001**	0.843	**<0.001**
**MoCA Total**	0.858	**<0.001**	-	-	0.768	**<0.001**
**MoCA Delayed Recall Score**	0.843	**<0.001**	0.768	**<0.001**	-	-
**ACE-R Total Score**	0.841	**<0.001**	0.871	**<0.001**	0.731	**<0.001**
**ACE-R Attention**	0.695	**<0.001**	0.696	**<0.001**	0.580	**<0.001**
**ACE-R Memory**	0.774	**<0.001**	0.826	**<0.001**	0.685	**<0.001**
**ACE-R Verbal Fluency**	0.613	**<0.001**	0.674	**<0.001**	0.522	**<0.001**
**ACE-R Language**	0.620	**<0.001**	0.613	**<0.001**	0.502	**<0.001**
**ACE-R Visual–executive**	0.591	**<0.001**	0.657	**<0.001**	0.509	**<0.001**

r: Spearman correlation coefficient.

**Table 5:** Areas under the curve obtained for clinical values.

Variables	AUC (95% CI)	Youden Index	Cut-Off Point	*p*	Sensitivity (%)	Specificity (%)
**MMSE**	0.926 (0.890–0.962)	0.749	≤25.5	**<0.001**	82.70	92.20
**MoCA Total**	0.946 (0.917–0.976)	0.775	≤22.5	**<0.001**	86.40	91.10
**MoCA Delayed Recall Score**	0.886 (0.840–0.931)	0.602	≤2.5	**<0.001**	62.40	97.80
**ACE-R Total Score**	0.967 (0.939–0.994)	0.900	≤68.5	**<0.001**	90.00	100.00
**ACE-R Attention**	0.807 (0.743–0.872)	0.559	≤13.5	**<0.001**	63.60	92.20
**ACE-R Memory**	0.997 (0.992–1.000)	0.991	≤15.5	**<0.001**	99.10	100.00
**ACE-R Verbal Fluency**	0.872 (0.819–0.925)	0.743	≤7.5	**<0.001**	75.50	98.90
**ACE-R Language**	0.729 (0.657–0.801)	0.515	≤16.5	**<0.001**	58.20	93.30
**ACE-R Visual–executive**	0.780 (0.714–0.846)	0.541	≤11.5	**<0.001**	56.40	97.80

**Table 6:** Distribution of MMSE, MoCA Total, and ACE-R Total Score cut-off points according to cranial MRI-phase status.

	Cranial MRI-Fazekas		
	I	II	III
	*n* (%)	*n* (%)	*n* (%)
**MMSE**			
≤25.5	13 (61.9)	34 (85.0)	44 (89.8)
>25.5	8 (38.1)	6 (15.0)	5 (10.2)
**MoCA Total**			
≤22.5	19 (90.5)	34 (85.0)	42 (85.7)
>22.5	2 (9.5)	6 (15.0)	7 (14.3)
**ACE-R Total Score**			
≤68.5	18 (85.7)	33 (82.5)	48 (98.0)
>68.5	3 (14.3)	7 (17.5)	1 (2.0)

## Data Availability

The data presented in this study are available on request from the corresponding author. The data are not publicly available due to privacy and ethical restrictions.
